# Insomnia in neurological diseases

**DOI:** 10.1186/s42466-021-00106-3

**Published:** 2021-03-10

**Authors:** Geert Mayer, Svenja Happe, Stefan Evers, Wiebke Hermann, Sabine Jansen, Ulf Kallweit, Maria-Lucia Muntean, Dieter Pöhlau, Dieter Riemann, Michael Saletu, Melanie Schichl, Wolfgang J. Schmitt, Friederike Sixel-Döring, Peter Young

**Affiliations:** 1 Neurologische Abteilung der Hephata-Klinik, Schimmelpfengstrasse 6, 34613 Schwalmstadt-Treysa, Germany; 2 Neurologische Abteilung der Philipps-Universität Marburg, Mamburg, Germany; 3 Klinik Maria Frieden, Klinik für Neurologie, Am Krankenhaus 1, 48291 Telgte, Germany; 4 Krankenhaus Lindenbrunn, Abteilung Neurologie, Lindenbrunn 1, 31863 Coppenbrügge, Germany; 5 Klinik und Poliklinik für Neurologie und Deutsches Zentrum für Neurodegenerative Erkrankungen e.V. (DZNE), Gehlsheimer Str. 20, 18147 Rostock, Germany; 6 Deutsche Alzheimer Gesellschaft e.V. Selbsthilfe Demenz, Friedrichstr. 236, 10969 Berlin, Germany; 7 Klin. Schlaf- und Neuroimmunologie, Private Universität Witten/Herdecke gGmbH, Alfred-Herrhausen-Str. 50, 58448 Witten, Germany; 8 Paracelsus Elena Klinik, Schanzenstr. 85 Dr. med Dieter Pöhlau, 34130 Kassel, Germany; 9 DRK Kamillus Klinik, Hospitalstr. 6, 53567 Asbach, Germany; 10 Psychiatrische Universitätsklinik Freiburg, Hauptstraße 5, 79104 Freiburg, Germany; 11 LKH – Graz II, Standort Süd, Wagner Jauregg Platz 1, A-8053 Graz, Austria; 12 Universitätsklinik für Psychiatrie und Psychotherapie, Murtenstrasse 21, 3008 Bern, Switzerland; 13 Paracelsus-Elena-Klinik, Klinikstr. 16, 34128 Kassel, Germany; 14 Neurologische Klinik Reithofpark, Reithof 1, 83075 Bad Feilnbach, Germany

**Keywords:** Insomnia, Neurological diseases, Comorbid diseases, Classifications, Diagnostic instruments, Therapeutic recommendations, Cognitive behavioral therapy

## Abstract

Insomnia is defined as difficulties of initiating and maintaining sleep, early awakening and poor subjective sleep quality despite adequate opportunity and circumstances for sleep with impairment of daytime performance. These components of insomnia – namely persistent sleep difficulties despite of adequate sleep opportunity resulting in daytime dysfunction - appear secondary or co-morbid to neurological diseases. Comorbid insomnia originates from neurodegenerative, inflammatory, traumatic or ischemic changes in sleep regulating brainstem and hypothalamic nuclei with consecutive changes of neurotransmitters. Symptoms of neurological disorders (i.e motor deficits), co-morbidities (i.e. pain, depression, anxiety) and some disease-specific pharmaceuticals may cause insomnia and/or other sleep problems.

This guideline focuses on insomnias in headaches, neurodegenerative movement disorders, multiple sclerosis, traumatic brain injury, epilepsies, stroke, neuromuscular disease and dementia.

The most important new recommendations are: Cognitive behavioral therapy (CBTi) is recommended to treat acute and chronic insomnia in headache patients. Insomnia is one of the most frequent sleep complaints in neurodegenerative movement disorders. Patients may benefit from CBTi, antidepressants (trazodone, doxepin), melatonin and gaba-agonists. Insomnia is a frequent precursor of MS symptoms by up to 10 years. CBTi is recommended in patients with MS, traumatic brain injury and. Melatonin may improve insomnia symptoms in children with epilepsies. Patients with insomnia after stroke can be treated with benzodiazepine receptor agonists and sedating antidepressants. For patients with dementia suffering from insomnia trazodone, light therapy and physical exercise are recommended.

## Introduction

Insomnias with difficulties of initiating and maintaining sleep, excessive daytime sleepiness, motor disorders during sleep and parasomnias, early awakening and impaired sleep quality frequently accompany neurological diseases as secondary or comorbid conditions. The underlying causes of many insomnias have not been fully elucidated, yet. Secondary insomnia may originate from neurodegenerative, inflammatory, traumatic or ischemic damage in sleep regulating brainstem and hypothalamic nuclei with consecutive changes of neurotransmitters. Symptoms of neurological disorders (i.e. motor deficits), co-morbidities (i.e. pain, depression, anxiety) and some specific medication result in insomnia and/or other sleep problems. Insomnias have a strong impact on quality of life, cognition and physical well-being and therefore need special consideration for diagnosis and therapy.

This guideline focuses on insomnias in headaches, neurodegenerative movement disorders, multiple sclerosis, traumatic brain injury, epilepsies, stroke, neuromuscular disease and dementia.

## Classification and definitions

The ICD 10/11, ICSD 3 and DSM V differentiate as “transient” or “chronic” insomnia. The ICD 11 contains a special chapter on sleep disorders, in which insomnia disorder is differentiated into short-term, chronic and unspecified insomnia disorders.

Chronic insomnia disorder is defined by the following diagnostic criteria (ICD 10):

A. Patient reports or caretaker observes
  1. Difficulty initiating sleep
  2. Difficulty maintaining sleep
  3. Waking up earlier than desired
  4. Resistance to going to bed on appropriate schedule
  5. Difficulty sleeping without parent or caregiver intervention
B. The sleep disorder causes significant clinical suffering or impairment in social-, educational- and professional life or other important domains
C. The sleep disturbance and associated daytime symptoms occur at least three times per week
D. The sleep disturbance and associated daytime symptoms have been present for at least 3 months
E. The sleep disorder occurs despite sufficient opportunity to sleep
F. The insomnia is not attributed more clearly by another sleep disorder
G. The insomnia is not caused by the physiological effects of a substance
H. The co-existing psychological and physical diseases do not explain the insomnia. If criteria for insomnia are met in context with a psychological or physical disease, both disorders will be diagnosed

In contrast to situational and transient insomnias defined as insomnia symptoms attributed to a certain event/situation or occurring transiently (meaning less than 3 months), occurring frequently without the need of specific interventions, chronic insomnias require specific treatment due to its impact on sleep quality, daytime performance and quality of life. According to ICD-10 [1] a disease-worthy insomnia is given, when symptoms persist for 4 weeks. According to DSM-5 [2] disease duration of 3 months is required, which is then called chronic insomnia. Insomnia in neurological diseases is also classified according to ICD-10 [1].

## Diagnostics

According to ICD 10/11 [1] the diagnosis of insomnia is based on clinical evaluations. Questionnaires may be added to complete evaluation of clinical symptoms within the diagnostic workup. For some of the neurological diseases, specific questionnaires addressing insomnia/sleep disturbances were designed, which are cited in the respective chapters. The recommended diagnostic work-up steps are as follows:

- Medical history/diagnostic work-up
- Psychiatric/psychological history
- Sleep history
- Actigraphy
- Polysomnography

### Recommendations

- The diagnostic work-up should comprise a complete medical history including physical and mental diseases, a physical examination, sleep questionnaires and sleep diary.
- The use of substances known to compromise sleep quality should be assessed thoroughly.
- Actigraphy may be used to evaluate bed and sleep times across 24 h and for longer time periods of days and weeks to assess circadian rhythm
- Polysomnography should be performed in cases suspicious for underlying organic sleep disorders (e.g., periodic leg movements, sleep related breathing disorders)
- Polysomnography is recommended
  - in therapy-refractory insomnia
  - after the above mentioned diagnostic steps to rule out secondary causes for insomnia have been performed
  - if insomnia potentially endangers patients themselves or others
  - in case of discrepancies between the subjective perception of insomnia and clinical and objective evaluations

## Therapeutic principles

4.1. In general, patients sleep hygiene should be evaluated. Informations on improving sleep hygiene (psychoeducation) should be conveyed. Strategies to improve day-structuring should be considered.

4.2. Flow chart

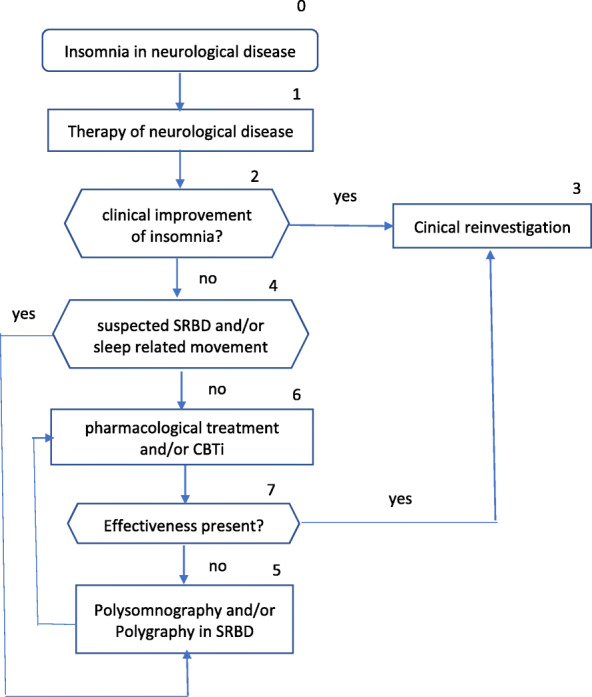



Insomnias comorbid to neurological disease should be treated according to international insomnia guidelines.

Collaboration with a certified sleep specialist is recommended for diagnostic work-up and treatment of insomnias. If polysomnography is indicated, the sleep laboratory should be accredited by the national sleep society and should be experienced in diagnosis and treatment of insomnias.

### Insomnia in neurological diseases

The following chapter presents insomnias in the most common neurological diseases. Insomnia in restless legs syndrome and narcolepsy are covered in the respective European and American guidelines.

### Insomnia in headaches

The International Headache Society (IHS) differentiates more than 200 forms of headaches in the International Classification of Headache Disorders, which are mainly unrelated to sleep. However, sleep related headaches usually emerge during sleep and may be accompanied by insomnia (according to ICSD3 [3–5]). These include different types of headaches such as migraine, cluster headache, chronic paroxysmal hemicrania (CPH) and hypnic headache syndrome. All of these forms show a close relationship between onset of pain and sleep and are listed separately in appendix A of the ICSD3 (ICSD-32014).

In a large Korean population-based evaluation, migraine was reported in 46% and other headache forms in 33% of patients with impaired sleep duration, whereas only 20% of controls suffered from headaches [6]. Similarly, migraine frequency increased in patients with short compared to longer sleep duration [7, 8].

In polysomnography study about 50% of patients with chronic tension headache and chronic migraine insomnia reported insomnia, but objective sleep parameters did not differ significantly from those without insomnia [9]. Interestingly, patients with the diagnosis of tension headache showed an increased amount of slow wave sleep with only few arousals in a Norwegian polysomnography based study, [10]. In an American population-based evaluation, insomnia was associated with overuse of medication in 44% of headache patients [11].

In a pilot trial 31 patients with either chronic insomnia or co-morbid (secondary) insomnia received either chronic behavioral therapy (CBTi) twice per week for 30 min (including advice on combined sleep hygiene and sleep restriction regimen) or unspecific advice on life style adjustments [12]. After 2 weeks the Intention-to-Treat analysis showed a reduction of headache frequency of 26,9% in the CBTi group and 36,2% in the control group. The follow-up examination after 6 weeks of treatment showed a frequency reduction of headache episodes of 48% in the CBTI group and 25% in the control group. Significant improvement of objective sleep efficiency as assessed by actimetry as well as subjective sleep quality was detectable in the CBTi condition compared to the control group. In a controlled,double-blind pilot trial (RCT) 79 patients with migraine were randomized 1:1 to receive either 3 mg eszopiclone or placebo at bedtime demonstrating significantly higher sleep efficiency and total sleep time as well as lower wake time frequency of nocturnal awakenings in the eszopiclone group [13].

#### Recommendations

- Besides symptom-oriented drug therapy, combined drug and CBT-I is recommended.
- Eszopiclone may be administered for at least 6 weeks in migraine patients with insomnia, since it does not have any influence on frequency, duration and intensity of migraine attacks.

### Insomnia in neurodegenerative movement disorders

The recommendations are restricted to Parkinson’s disease (PD), atypical Parkinson syndromes, and spinocerebellar ataxia (SCA).

Sleep disorders in neurodegenerative diseases affect up to 90% of all patients. Sleep disorders in neurodegenerative diseases can precede motor symptoms by years and may exhibit unfavorable impact on quality of life and cognition [14]. Insomnia is the most frequently associated sleep disorder [15]. 35–60% fulfill the criteria of chronic insomnia [15].

To screen for sleep problems and score their severity in PD the task force of the Movement Disorder Society (MDS) recommends the use of the following scales and questionnaires: Parkinson’s disease sleep scale (PDSS), Pittsburgh sleep quality index (PSQI) and SCOPA-Sleep [16].

Most studies on possible treatment options for insomnia in movement disorders have been performed in PD patients. Currently, there are no RCT treatment trials available addressing insomnia in supranuclear gaze palsy (PSP), corticobasal degeneration (CBD) or multisystem atrophy (MSA). The Task Force of the Movement Disorder Society for evidence-based medicine concludes that due to lack of studies no specific pharmacotherapy can be recommended for long-term treatment of sleep disorders in PD at present [17]. However, case series and small randomized studies report partially positive effects of some drugs. The European Federation of Neurological Societies/Movement Disorder Society for the therapy of insomnia in PD recommends the exclusion or otherwise treatment of other sleep disorders, and the identification and treatment of PD specific motor complications and symptoms [18].

In PD patients the amplitude of the rhythmic expression of clock genes such as per 2 as well as the transcription factor bmal 1 was reduced, supposedly influencing gene transcription and inflammatory processes. These changes of circadian processes might be a consequence of the ongoing neurodegenerative process but may also contribute to disease progression itself [19, 20]. Analyses of a national data banks in Great Britain and Taiwan showed a prodromal incidence risk of insomnia for clinical manifestation of PD of 1,38 (CI 1,11 – 1,70) [21, 22].

In a Finnish questionnaire-based evaluation in 1447 randomly chosen PD patients chronic insomnia was reported by 36,9% with sleep maintenance problems as the mostly prominent feature in 81%. Others reported a close correlation between low sleep quality, chronic insomnia and restless legs syndrome symptoms [23]. Insomnia in PD seems to be associated with age [24], disease duration, female sex, use of dopamine agonists [25] as well as neuropsychological symptoms such as depression/anxiety, even in de novo PD patients at an early disease stage [26, 27]. In a Dutch longitudinal, prospective, cohort-study insomnia was also associated with motor fluctuations, autonomic dysfunction and hallucinations [28].

Although dopamine agonists were associated with insomnia, extended-release formulations of dopamine agonists seem to improve subjective and objective sleep quality [29] but also motor symptoms [30, 31]. Furthermore, in small case series antidepressants (AD) (i.e. trazodone, doxepine, venlafaxine, nortryptyline), as well as melatonin / ramelteon, pimavanserine and GABA-agonists (eszopiclone/zolpidem), exerted a positive effect on insomnia symptoms in PD patients [32–34], whereas duloxetine, quetiapine and clozapine failed to show an improvement in sleep quality [32].

Small interventions studies on CBTi, sometime combined with light therapy, showed a significant improvement in subjective sleep quality [35, 36]. Effects of deep brain stimulation (DBS) on sleep quality are not fully elucidated, yet. While a few questionnaire-based case series on DBS of the subthalamic nuclei resulted in improvement of subjective sleep quality [37–39], a small pilot trial in 5 patients with DBS of the globus pallidus internus failed to show any significant improvement of polysomnographically assessed sleep efficiency and sleep latency as well as subjective sleep quality [40].

Apart from PD, sleep disturbances also occur in other movement disorders. In atypical Parkinson syndromes, such as Multisystem atrophy (MSA), the reported incidence of insomnia is about 70% compared to 62% in PD and also in Progressive supranuclear palsy (PSP) insomnia and disturbed sleep architecture occurred more frequently than in PD (Abbott 2014, [41]). Also, 37,7% of patients with SCA type 3 suffer from insomnia [42], while a case series of patients from one family with SCA type 2 showed a strong association between insomnia and depression [43].

#### Recommendations

- Treating with or switching to extended-release dopaminergics, either as transdermal application or extended-release levodopa, is recommended to improve subjective sleep quality.
- Eszopiclone, doxepine, zolpidem, trazodone, ramelteon and melatonin can be used for the treatment of insomnia in PD patients. However, evidence for the efficacy is insufficient.
- Subjective sleep quality can be improved by treatment with the antipsychotic drug pimavanserine and the antidepressants venlafaxine and nortriptiyline. Treatment with duloxetine, quetiapine and clozapine for insomnia is not recommended due to insufficient evidence of effectiveness.
- Bright light therapy with 1000–7500 lx for 30–90 min can be recommended for the treatment of insomnia in PD.
- Optimizing sleep hygiene, CBTi, light therapy and melatonin can be used for the treatment of insomnia in PD
- Consider multifactorial causes of insomnia: comorbidities such as RLS, PLMS, re-emergence of motor or non-motor symptoms like akinesia, rigor und/or resting tremor and non-motor symptoms and complications like hallucinations should be identified by adequate diagnostic procedures and addressed specifically.
- Sleep quality of caretakers should also be evaluated since their sleep disorders may lead to impaired quality of care.

### Insomnia in multiple sclerosis

Multiple sclerosis (MS) is frequently associated with sleep disorders: Insomnia (25–55%), RLS (5–19%) and sleep related breathing disorder (SRBD, 20–60%). The causes for insomnia are manifold, and include primary insomnia, pain, nocturia, spasticity and obesity. Some specific MS drug regimens can contribute to insomnia. Insomnia is associated with physical and functional impairment and depression. Depending on criteria and type of assessment 25–54% of MS patients suffer from non-restorative sleep [44–46].

At MS onset sleep quality in younger adults is mainly normal [111]. The risk to develop insomnia is associated with increased fatigue scores [47]. A prospective multicenter study in Portugal including 206 MS patients, detected chronic insomnia in 22,6%, frequently associated to female sex, anxiety disorder, fatigue and presence of other comorbidities [48]. Others described a prevalence of insomnia varying between 13,2% of MS patients with relapsing and remitting MS (RRMS) [49] and 12,5% with about half of them suffering from RRMS [50]. In 10,000 MS patients and 40,000 matched controls from a database insomnia was identified as a prodromal symptom of MS occurring up to 10 years prior to disease onset [51]. MS patients with insomnia showed a higher arousal level than patients with insomnia only or patients with other neurological disorders [52]. A Dutch study (71 MS patients, 40 controls) showed that in MS patients with sleep disturbances cognitive impairment was pronounced and thalamic functional connectivity was impaired [53].

Data on the effect of disease modifying therapies on sleep and fatigue are scarce. However, some studies describe symptom improvement after or during treatment with disease modifying agents [47, 54]. On the contrary, in patients with highly active RRMS treated with monthly intramuscular injections of ACTH insomnia was one of the most frequent side effects [55]. However, most studies on insomnia in MS are questionnaire-based and do not take disease severity or drug regimens into account.

A small North American study showed efficacy of CBTi on insomnia symptoms in MS [56], another small study showed decreased melatonin levels after steroid pulse therapy in an MS animal model and in MS patients [57]. Besides, in a case control study with 202 MS patients 5 mg of melatonin exerted positive effects on insomnia symptoms [58].

#### Recommendations

- CBTi is recommended despite insufficient data from RCTs.
- Melatonin is recommended despite insufficient data from RCTs.
- ADs are recommended in MS patients with comorbid depression.
- Diagnostic work-up of insomnia is recommended before initiating specific therapies.
- As insomnia often occurs prior to MS onset, MS should be considered in the differential diagnosis of insomnia.
- The association between fatigue and insomnia and the differentiation of both symptoms should be considered in the diagnostic and therapeutic process.

### Insomnia in traumatic brain injury

Traumatic brain injury (TBI) is considered as the most frequent cause for persisting neurological handicap.

More than 50% of TBI patients suffer from sleep-wake disorders independent of the severity of TBI. Insomnia symptoms exert negative influences on neuroplasticity and the recovery process, as well as on cognition and nocturnal pain sensation and lowers the threshold for psychiatric comorbidity.

A meta-analysis of 1706 participants suffering from TBI of all degrees of severity identified insomnia in 29%, hypersomnia in 28% and sleep apnea in 25% of patients [59]. In 95% of TBI patients decreased CSF-hypocretin-1 levels have been demonstrated during the acute phase. These findings seem to be persistent mainly in patients with a narcolepsy-like phenotype. Congruently, post mortem studies revealed a reduction of hypothalamic hypocretin neurons in TBI patients (Review: [60]). Furthermore, more recent post mortem studies from the same group showed a 41% reduction of tuberomammilary histaminergic neurons in seven out of 12 patients, but only a 21% reduction of hypocretin neurons [61]. Female gender, impaired pre-traumatic sleep quality, reduced sleep perception and cognition during the first 2 weeks after acute TBI are predictive for chronic insomnia [62]. Circadian sleep-wake-rhythm disorders, especially the “delayed sleep phase syndrome” are considered as an important differential diagnosis in more than a third of all TBI patients suffering from insomnia [63]. In 42% of all TBI patients reduced melatonin levels in their evening saliva probes anda delayed nocturnal melatonin synthesis were detected [64]. Patients with severe TBI showed an increase of slow wave sleep proportion during their chronic phase, suggesting an increase in posttraumatic neuroplasticity [63, 65]. No RCTs were identified specifically addressing treatment of insomnia in TBI.

#### Recommendations

- Symptom oriented somatic (i.e. CPAP in obstructive sleep apnea) and pharmacological therapy is recommended taking comorbidities into consideration.
- Depending on the clinical conditions the combination of pharmacological therapy and CBTi should be considered.

### Insomnia in epilepsy

The prevalence of epilepsy in adults is about 3%. In a Chinese study including 150 adult patients with epilepsy (according to the International Classification of Epilepsies and Epileptic Syndrome) suffering from subjective sleep disorders compared to 100 healthy age- and sex matched controls, no differences regarding overall sleep quality using the Pittsburgh Sleep Quality Index (PSQI) were detected. However, the Insomnia Severerity Index (ISI) showed showed significantly higher scores in the epilepsy group. The ISI could identify insomnia in 19% of all epilepsy patients. Those epilepsy patients with insomnia were significantly more often treated with lamotrigine than those without insomnia [66]. In a prospective longitudinal study comprising 95 patients with unclassified epilepsies, 65,5% suffered from mild to moderate, and 28,9% from severe insomnia. Among the predictors for severe insomnia were a history of prior head trauma, previous epilepsy surgery, intake of sedative drugs, and antiepileptic polypharmacotherapy [67].

In a questionnaire-based evaluation comparing sleep quality and insomnia frequency in 208 epilepsy patients and 212 controls, 24% of epilepsy patients compared to 20% of controls suffered from chronic sleep initiation difficulties. Chronic insomnia was diagnosed in 36% of epilepsy patients and 15% of controls. Sleep quality assessed by the PSQI was classified as impaired in 30% of epilepsy patients and 16% in controls [68]. In a Spanish study 22% of 237 patients with non-refractory epilepsy suffered from sleep disorders compared to 45% out of 264 patients with refractory epilepsy [69].

In a double-blind, crossover, placebo-controlled study in in 11 children aged 6–11 years 9 mg slow release melatonin, taken 30 min prior to bed time, resulted in a significantly reduced sleep latency [70] In 165 American veterans with epilepsy the prevalence of insomnia was 40%. TBI, intake of lamotrigine and affective disorder showed an increased association with insomnia [71]. A non-controlled phase 3 study to evaluate the add-on therapy of brivaracetam on focal epilepsies found insomnia in 3% of the group treated with 20 mg brivaracetam, and 6,9% in the group treated with 50 mg [72].

A metaanalysis found a prevalence of 36–74.4% in adult epilepsy patients (mainly difficulties of maintaining sleep) and 11% in the pediatric population [73]. In a prospective study in nine patients deep brain stimulation of the anterior thalamic nucleus (ANT) was associated with polysomnographically detected awakenings in 14–67% of stimulations [74].

Of note, most studies were limited by sleep evaluation using questionnaires instead of polysomnography and epilepsy syndromes were not classified sufficiently.

#### Recommendations

- AE that do not exert influence on sleep should be preferred.
- PSG can be used in patients treated with deep brain stimulation to detect stimulus associated arousals.
- Slow release melatonin can be used in epilepsy to shorten sleep latency (although this has only been verified in children so far).

### Insomnia in neuromuscular disease

A German study compared the frequency of non-motor symptoms in 90 NMD patients with an age matched control group demonstrating significantly more insomnia symptoms in the NMD group [75]. In a Japanese case report insomnia was the leading complaint in a patient with ALS [76].

A Chinese study investigated the use of integrative therapies (chinese herbs, massage, acupuncture) in 258 patients with amyotrophic lateral sclerosis (ALS) and found a higher use of these therapies in ALS patients with insomnia than in those without [77]. An Italian study investigated the association of sleep disorders in facial-scapular-humeral dystrophy (FHSD). Polysomnographic data of 32 patients with a genetic form of FSHD were compared to those of an age matched control group. The FSHD group showed longer sleep latencies, shorter sleep duration, more frequent wake episodes and a shorter amount of REM sleep. With disease progression FSHD patients had less body movements in sleep [78].

An Italian and an American study describe each a case with Morvan syndrome. In both cases severe neuromyotonia was combined with severe insomnia, and in both cases antibodies against voltage-gated potassium channels were detected. In one of these cases plasmapheresis resulted in a restitution [79], in another case restitution was achieved by thymectomy [80].

An American study investigated the frequency of sleep disorders using polysomnographic data a in 8 patients with myotonic dystrophy type 2. Five (62,5%) patients suffered from insomnia [81].

An Italian study compared the frequency of SRBD in 13 patients with sporadic inclusion body myositis (IBM) to those of age matched controls and found significantly lower sleep efficiency, a higher frequency of nocturnal awakenings, and significantly longer nocturnal wake times [82].

Limitations: Very low patient numbers.

#### Recommendations

- Insomnia should be considered as a relevant sleep disorder in ALS.
- Insomnias should be taken into anamnestic and diagnostic considerations in FSHD, IBM and MD2.
- Insomnia must be considered as an integral sleep disorder in patients with Morvan-Syndome, which may improve by treatment with plasmapheresis or thymectomy.

### Insomnia in stroke

Several studies identified insomnia to be responsible for a mild to moderate increase of the risk for stroke, whereas other studies did not detect an increased risk. These discrepancies might partially be attributed to other confounding factors.

The risk for stroke was increased in patients with insomnia in a population based-study in a 10-year follow-up (HR 1.85; 1.62–2.12) after controlling for other risk factors [83]. A registry-based study with 21,430 patients with insomnia found an increased stroke risk in a four-year follow-up (HR 1.54; 1.38–1.72) [84]. A population-based study with 5494 patients found a slightly increased risk for a former stroke in patients with insomnia symptoms (OR 1.26; 1.03–1.55), which could not be confirmed at follow-up over up to 6 years [85]. A meta-analysis of prospective population-based studies found the risk for a later cardio-cerebral vascular event slightly increased in persons with difficulties of sleep initiation (RR 1.27; 1.15–1.40) and people with difficulties to maintain sleep (RR 1.11; 1.04–1.19) as well as non-restorative sleep (RR 1.18; 1.05–1.32), whereas the risk for stroke was not elevated in persons reporting early awakenings. The analysis did not discriminate between cardial and cerebral events [86]. Another population-based study did not find an increased risk for stroke in patients with difficulties initiating and maintaining sleep after controlling for other risk factors. Only in men with either short sleep (<=5 h) or long sleep duration (> = 10 h) a slight, but not significant, increase of stroke risk was detected after controlling for several risk factors (HR 1.89; 0.96–3.73 resp.HR 1.72; 0.89–3.33) [87].

The HUNT 3 study (*n* = 24,715) found no increased stroke risk in insomnia patients after correction for confounding variables [88].

### Insomnia after stroke

In a review insomnia was described in 50% of patients after stroke, of whom 1/3 did not suffer from insomnia prior to stroke. Symptoms are moderated by environmental factors and comorbidities, rarely as a result of brain damage [89]. In a prospective study 59.8% of patients (*N* = 214) experienced at least one symptom of insomnia in the first month after stroke [90]. In a longitudinal cohort study in (*n* = 100) women impaired sleep quality (as measured by the PSQI) was detected during the acute phase, but not after 6 months of follow-up [91]. A prospective study in 508 patients found symptoms of insomnia 3 months after stroke in 36.6% patients, in 12.6% with symptoms sequelae during daytime, mainly in those with stroke of the frontal lobe [92].

A multicentric, prospective study (*n* = 809) found insomnia according to DSM IV criteria in 26.9% of all patients and any type of sleep disorder in 56.7% [93]. A prospective multicenter study with 441 patients, age < 65 years found insomnia in 30–37% of all patients after 1 year of follow-up. 16% suffered from chronic insomnia after 6 months, and had an increased risk for feeling depressed (OR 6.75; 2.78–16.4), anxious (OR 3.31; 1.54–7.09) or to have a physical handicap (OR 3.60; 2.07–6.25) [94].

Quality of life in the first weeks after stroke was worse in patients with insomnia than in those without [95]. Health related quality of life HRQol was reduced in 336 patients with insomnia which occurred after stroke [96]. Symptoms of insomnia and reduced HRQol with physical and even more mental factors correlated in 214 patients 1 month after stroke [90].

Treatment of insomnia after stroke should comprise behavioral strategies such as a silent environment during the night with protection from too much light, during daytime much light exposure and physical activity. Drug treatment should comprise benzodiazepine receptor-agonists (zolpidem) or sedating antidepressants (amitriptyline, trazodone, mianserin, mirtazapine) [89]. A randomized controlled pilot-study in 15 patients found improved sleep disorder after 4 months of CBTi compared to unspecific treatment, which did not persist at follow-up [97]. A small prospective study in 30 patients with subacute stroke did not show differences of function, cognition and depression between stroke patients after stroke and patients with stroke and insomnia treated with hypnotics (*n* = 15 Zolpidem, in one patient additional 0.125 mg triazolam and in two patients trazodone 25 mg) after 3 weeks. This result indicates that hypnotics do not hamper rehabilitation [98]. Acupuncture has proven to be helpful in the treatment of insomnia after stroke [99].

#### Recommendations

- Since insomnia occurs frequently in acute stroke, screening for insomnia should be performed.
- Insomnia in stroke patients should be treated to improve the outcome of stroke and to improve quality of life.
- Therapies using bright light, benzodiazepine-receptor agonists, sedating antidepressants, CBTi and acupuncture can be recommended.

### Insomnia in dementia and prion-diseases

Literature of the past 20 years has given proof that sleep disorders of any kind may be one of the first signs or may even directly contribute to the development of dementias, with insomnia being of special interest for the development of Alzheimer dementia (AD). Early diagnosis and treatment of sleep disorders dementia could add to the prevention of dementias.

Aggregation of beta amyloid (Aß) starts about 20 years prior to diagnosis of AD. Short sleep duration seems to impair Aß elimination by the glymphatic system and seems to enhance production of Aß, leading to Aß accumulation in the brain.

A population-based Italian study found dementia in 86 of 750 persons > 65 years. Of those 84,7% had insomnia, 26,2% snoring and apneas, 25.7% leg movements, 30,6% excessive daytime sleepiness (eds)). EDS was the only predictor for cognitive decline [100].

In a crosssectional questionnaire-based evaluation in 500 patients with early forms of Lewy body dementia (DLB), Alzheimer (AD) and PD dementia (PDD) detected insomnia in 29,9%, nocturnal cramps in 24,1%, eds in 22,6%, RLS in 20,7% and RBD in 18,5% of patients. Patients with AD reported lower frequencies of sleep disturbances compared to DLB and PDD [101].

Patients suffering from long lasting insomnia aged 50–65 years have a higher risk to develop dementia (HR, 5.22; 95% CI, 2.62–10.41) than patients > 65 years without insomnia. Use of hypnotics with long half-life and high doses of hypnotics increase the risk for dementia [102]. In persons > 75 years difficulties to maintain sleep increased the risk for cognitive decline of previously cognitively not impaired persons [103].

Sleep disorders with prolonged sleep latency and low sleep efficiency were associated with cognitive frontal executive and visuospatial dysfunctions [104].

Polysomnographies of patients with Creutzfeldt-Jakob disease showed changes of the sleep structure: Reduction of total sleep time, sleep spindles and K complexes as well as sleep fragmentation and REM sleep without atonia. Comorbid sleep disorders in these patients are hypersomnia in 39%, insomnia in 46%, RLS in 25% and parasomnias in 32%. Patients can benefit from treatment of their comorbid sleep disorders [105].

A combination of bright light exposition and walking exercise for > 4 days/week increased sleep duration measured by actigraphy in AD patients [106].

In an evidence-based guideline for “ Prescribing antipsychotics for behavioural and psychological symptoms of dementia and insomnia” (VSPD) recommendations on the handling of insomnia symptoms are given: 1. in patients, in which symptoms are stabilized or who show no response to adequate treatment for 3 months, antipsychotics should be slowly tapered every 1–2 weeks in collaboration with the patient and the caregiver (strong recommendation, moderate evidence). This recommendation is not applicable to psychotic patients 2. For adults with primary insomnia treated for any duration or secondary insomnia, in which underlying comorbidities are managed, antipsychotics can be stopped; tapering is not needed (good practice recommendation) [107].

A Cochrane meta-analysis (2013) including 10 studies showed little or no evidence, that tapering of neuroleptics that had been administered over a long time period in patients with VSPD older than 65 years had any effect on the symptoms [108].

A 2016 Cochrane analysis [109] on four RCTs with melatonin administration of up to 10 mg and slow release melatonin of 2.5 mg over 8–10 weeks in patients with dementia and sleep disorders did not show significant improvement for any of the sleep parameters. In 30 patients treated with trazodone sleep duration and sleep efficiency increased, but there was no improvement in nocturnal wake time and frequency of awakenings. Trazodone 8 mg administered over 8 weeks did not show any effect on sleep parameters.

Limitations: Most studies assessed sleep disorders with sleep questionnaires, interviews or actigraphy. Few studies are RCTs. All of the studies used different protocols over different time periods. The recommendations are therefore of very low evidence and do not imply that certain treatments may not be helpful or efficacious.

#### Recommendations

- In patients with behavioral problems caused by dementia, whose insomnia problems have been treated insufficiently with neuroleptics over years, neuroleptics should be tapered.
- No benefit for the treatment of insomnia in demented patients could be shown for hypnotics such as benzodiazepines, benzodiazepine receptor agonists and phytotherapy. These drugs should not be administered in demented patients with insomnia in general.
- Immediate release, slow release melatonin and melatonin-agonists may be options in the treatment of insomnia patients with AD.
- Trazodone 50 mg at night can be recommended since it improves sleep duration and sleep onset time in patients with AD, but there is no evidence, that it improves sleep fragmentation.
- Bright light therapy (2500 Lux full spectrum) in combination with 30 min of walking on at least 4 days per week can be recommended to improve sleep duration.

## Financing

The travelling cost of participants were covered by DGN. The guideline was not sponsored.

## Methods of guideline development

### Participants from medical societies

For the German Society for Psychiatry and Psychotherapy DGPPN and for the German Society for Sleep Medicine: Prof. Dr. rer. Soc. Riemann.

For the German Society of Neurologists DGN and for the German Society for Clinical Neurophysiology and Functional Imaging DGKN Prof. Dr. med. Mayer.

German Alzheimer Society (Deutsche Alzheimer Gesellschaft e.V., Selbsthilfe Demenz): Mrs. S. Jansen, Berlin.

German Multiple Sclerosis Society (DMSG Bundesverband): Dr. D. Pöhlau, Aasbach.

German Parkinson Society (Deutsche Parkinson Gesellschaft e.V.): Dr. med. Wiebke Hermann, Rostock.

German Epilepsy Union (Deutsche Epilepsievereinigung): Participation was not possible.

Germain Migraine and Headache Society (Deutsche Migräne- und Kopfschmerzgesellschaft e.V.): Prof. Dr. med. Stephan Evers, Coppenbrügge.

German Stroke Society (Deutsche Schlaganfallgesellschaft): participation was not considered to be necessary.

### Participation of patient groups

Deutsche Alzheimer Gesellschaft e.V., Selbsthilfe Demenz, Friedrichstr. 236, 10,969 Berlin.

Deutsche Multiple Sklerose Gesellschaft, Bundesverband e.v. DMSG, Krausestr. 50, 30,171 Hannover.

Deutsche Parkinson Vereinigung - Bundesverband - e.V., Moselstraße 31, 41,464 Neuss.

Deutsche Epilepsievereinigung e.V., Bundesgeschäftsstelle, Zillestraße 102, 10,585 Berlin.

Deutsche Migräne- und Kopfschmerzgesellschaft, Migräne- und Kopfschmerzklinik Königstein; Ölmühlweg 31, 61,462 Königstein im Taunus.

### Search and selection of scientific literature

Search used medline, pubmed, embase, web of science und Cochrane database. Search included years 11/2009–12/2018 and was restricted to adult population. Search terms were “insomnia in neurologic disease”, “insomnia in central neurologic diseases”, and „insomnia and – respective neurological diseases”).

Articles had to contain at least one definition of insomnia according to recent classification systems. All randomized, controlled studies with > 5 patients were included. If studies with high evidence were lacking, studies of lower evidence were included, if they met the selection criteria. Evidence classification (class 1-!V) was perfomed according to standardized European Procedures [110]. Levels of recommendation are A-D. Literature was categorized independantly by two experts according to Oxford Centre for Evidence-based Medicine Levels of Evidence (2001).

### Consensus methods

Modified Delphi procedure: PD Dr. Sitter.

Consensus was reached with a nominal group process and delphi technique in two consensus conferences.

## Data Availability

N.A.

## Appendix

Links to sleep questionnaires

Pittsburg Sleep Quality Index https://www.outcometracker.org/library/PSQI.pdf

SF-A: https://www.researchgate.net/publication/285321057_Schlaffragebogen_A_und_B

Abend Morgen Protokoll: https://www.dgsm.de/downloads/fachinformationen/frageboegen/2wochen.pdf

Insomnia Severiy Index: https://deploymentpsych.org/system/files/member_resource/Insomnia%20Severity%20Index%20-ISI.pdf
